# Do Audible Sounds during a Lumbar Spine Thrust Manipulation Have an Impact on Brainwave Activity?

**DOI:** 10.3390/healthcare12171783

**Published:** 2024-09-06

**Authors:** Rob Sillevis, Tiffanny de Zayas, Anne Weller Hansen, Halle Krisinski

**Affiliations:** Department of Rehabilitation Sciences, Florida Gulf Coast University, Fort Myers, FL 33965, USA; tiffdezayas@gmail.com (T.d.Z.); awhansen@fgcu.edu (A.W.H.); hnkrisinski7932@eagle.fgcu.edu (H.K.)

**Keywords:** audible sounds, lumbar thrust manipulation, brainwave activity, EEG

## Abstract

Background: To manage pain and stiffness of the lumbar spine, thrust manipulation is commonly used. High-velocity, small-amplitude thrust manipulation often elicits audible sounds. What causes this audible sound remains unclear, and its clinical significance has not been shown. This study aimed to identify how audible sound affects brainwave activity following a side-lying right rotatory thrust manipulation in a group of healthy individuals. Methods: This was a quasi-experimental repeated measures study design in which 44 subjects completed the study protocol. A portable Bluetooth EEG device was used to capture brainwave activity. The environment was controlled during testing to minimize any factors influencing the acquisition of real-time EEG data. After a short accommodation period, initial brainwaves were measured. Following this, each subject underwent a lumbar 4–5 side-lying right rotatory thrust manipulation, immediately followed by a second brainwave measurement. A third measurement took place one minute later, followed by a fourth one at the three-minute mark. Results: 21 subjects did not experience audible sounds, while 23 subjects experienced audible sounds. Both groups had significant changes measured by the 14 electrodes (*p* < 0.05). The audible group had more significant changes, which lasted only two minutes. Conclusion: The lack of brainwave response differences between the audible and non-audible groups implies no direct, measurable placebo or beneficial effect from the audible sound. This study could not identify a benefit from the audible sound during an HVLA manipulation of the subjects.

## 1. Introduction

Spinal joint manipulation (SM) is an effective intervention for managing low back pain (LBP) [1,2]. High-velocity, low-amplitude (HVLA) thrust manipulations are the most used form of SM applications [3,4]. During an HVLA manipulation, a quick stretch of the joint capsule occurs. This stretch often accompanies an audible sound, typically perceived by the subject and the manipulator. Traditionally, practitioners performing HVLA manipulations use audible sound as a manipulation success indicator [4,5]. It has been reported that an audible sound occurs during an HVLA manipulation in 65% of cases [4]. The true clinical and physiological contribution of audible sounds remains elusive. Audible sounds have not been proven to affect clinical outcomes significantly [6,7].

Several concepts have been proposed to explain the phenomenon of audible sounds. Cavitation is one theory that claims that there is a gas release from joint synovial fluid when the joint is moved quickly at a short amplitude at the joint’s end range, which could result in an audible sound [8]. On the other hand, Brodeur [9] proposed that cavitation sounds result from the elastic recoil of the synovial capsule away from joint space during HVLA manipulations. Brodeur’s [9] consideration of the origination and benefit of the cavitation sound includes a neurological reflex reaction. Others have suggested that audible pops are due to ligamentous recoil [10]. However, the most prominent theory is the mechanism of viscous adhesion, or tribonucleation [8]. Tribonucleation occurs when two surfaces separated by a thin-fluid film are pulled apart rapidly. During the rapid manipulation, negative tension is created through the synovial fluid to resist bone separation. This negative pressure releases carbon dioxide from the synovial fluid, causing a short-term extension of the joint capsule, possibly accompanied by an audible sound [8].

Bialosky et al. [11] demonstrated neurophysiological effects following an HVLA manipulation, resulting in an audible pop. A generalized neurophysiological effect from HVLA manipulation is supported by the findings of Sillevis et al. [12], who reported an overall generalized increase in the magnitude of Theta brainwave activity in the bilateral parietal lobes and left occipital lobe, which indicates a more relaxed state. Despite the findings of hypoalgesia after HVLA manipulation, Flynn et al. [7] demonstrated no significant difference in joint range of motion, pain, and disability when audible sounds were elicited. Sillevis et al. [4] demonstrated that audible sounds accompanying a thoracic manipulation did not affect the autonomic nervous system. Therefore, currently, there is no support for the hypothesis that the audible pop accompanying an HVLT manipulation has any clinical relevance.

Even though the true benefit of audible sounds during HVLT manipulation remains questionable, both the subject and the practitioner are biased toward using audible sounds as a criterion for success. A post-manipulation change should occur if a relationship exists between audible sounds and the central nervous system. Central nervous system activity and immediate changes can be captured using electroencephalography (EEG). Electroencephalography captures real-time brainwave activity [13]. An EEG device consists of electrodes that are placed on the scalp. These electrodes measure electrical conduction and potentials generated in the brain. Combining the readings of numerous electrodes on the scalp makes readings of activity in the entire brain accessible to researchers [13]. Over the last few years, low-cost, consumer-grade (LCCG) headsets have been developed. The advantage of LCCG headsets is that they allow more mobility, are more affordable, and are more practical to use in research. Willems et al. [14] validated the LCCG-Emotiv EPOC+ device (Emotiv, San Francisco, CA, USA) compared to a medical-grade unit.

The Emotiv EPOC+ consists of a 14-lead EEG unit [14]. Brainwaves are categorized by their shape, amplitude, frequency, and the location of the leads they appear on [14]. Brainwaves are associated with the activity of specific brain regions. There are five main categories of brainwaves based on frequency. Delta waves, representing the brain’s gray matter, are found in the frequency range of 0.1 to 4 Hz and have the highest amplitude and the slowest wavelength. Delta waves are found in all stages of sleep but are abnormal in awake adults [14]. Theta waves emit frequencies of 4 to 8 Hz. These waves are correlated to subconscious activities and are typically found in states of deep relaxation, such as meditation. Theta waves contribute to the production of serotonin, human growth hormone, and cortical hormone; modulate pain; increase relaxation; and help facilitate memories and learning [15]. Alpha waves are found in frequencies of 8 to 13 Hz. They are typically recorded in the occipital and parietal regions on both sides of the brain. Alpha waves are commonly found in awake adults who are relaxed with closed eyes. Alpha waves also contribute to the production of serotonin, which increases relaxation and modulates pain. Alpha waves represent the white matter of the brain [15]. Beta waves emit frequencies of 13 to 30 Hz. These waves are associated with conscious activity and activity on both sides of the parietal and frontal lobes. Beta waves are present during conscious thinking and encourage cortisol production, impacting memory and learning [15]. Gamma waves include brainwave frequencies of 30 to 100 Hz. They are associated with consciousness and perception. They appear during hyper-alertness and the combination of sense and memory [15]. 

In summary, no current evidence exists that audible sounds generated during an HVLT manipulation influence clinical outcomes or directly affect the central nervous system. Therefore, this study evaluated whether there are any significant changes in brainwave activity following a side-lying HVLA manipulation of the lumbar spine when comparing the presence or absence of an audible sound in asymptomatic subjects.

## 2. Material and Methods

This quasi-experimental study used a method of convenience sampling with a within-subject repeated measures design. Subjects were recruited using a method of convenience in the fall of 2022 from the faculty, staff, and students at Florida Gulf Coast University (Florida, USA). Institutional review board (IRB# 2020-64 date: 3 August 2021) approval was obtained from Florida Gulf Coast University. This study was also registered at ClinicalTrial.gov with ID# NCT04542707. All subjects provided written consent prior to participating in the study. 

A total of 44 asymptomatic healthy subjects were recruited. Subjects were screened for the eligibility criteria. The subjects had to be between 18 and 65 years old to be included in this study. To give proper informed consent, the subjects had to be able to read English at the 12th-grade level. Exclusion criteria included a history of lower back injury in the preceding six months, a history of osteopenia or osteoporosis, or cancer. A history of brain injury (such as concussion or traumatic brain injury), possibly resulting in atypical neural signaling, was an additional exclusion criterion.

### Study Protocol

All testing was performed in the same room, where the temperature was constant, there was minimal electrical interference, and the light was off during testing, with only natural light present. This controlled location would have minimal interference with EEG data collection. Each subject entered the testing room after providing consent. Subjects were seated and researcher I applied the Emotiv EPOC+ headset. A saline solution optimized contact between the electrodes and the skull. Bluetooth wirelessly connected the Emotiv EPOC+ headset to the processing Emotiv Pro software (version 4.3.7.522). Brainwave data were collected concurrently in the 14 active electrodes with a frequency of 128 Hz. The Emotiv EPOC+ has been previously validated [15,16,17]. The 14 electrodes of the headset are placed on the head so that they follow the international 10–20 system for EEG measures [18]. The EPOC+ measures 5 different types of brainwaves: Theta waves at a frequency of 8–13 Hz, Alpha waves at 8–13 Hz, Beta low (also called Beta one) waves at 13–15 Hz, Beta high waves at 18–40 Hz, and Gamma waves at a frequency of 40–100 Hz. To prevent the development of spikes in brain activity not caused by the experiment, the EEG measurement protocol was time-based (15 s) for each measure. It was believed that this methodology allowed for better identification of changes in brain activity.

**Figure 1:**
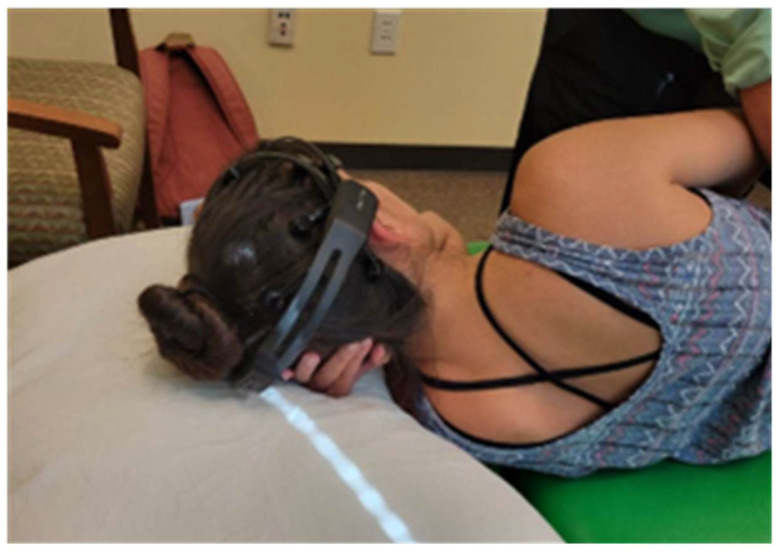
Participant in a side-lying position with the Emotiv EPOC+ in place.

After adequately applying the Emotiv EPOC+ and verifying connectivity through the Emotiv system, the subject was placed in a left side-lying position (Figure 1). 

**Figure 2:**
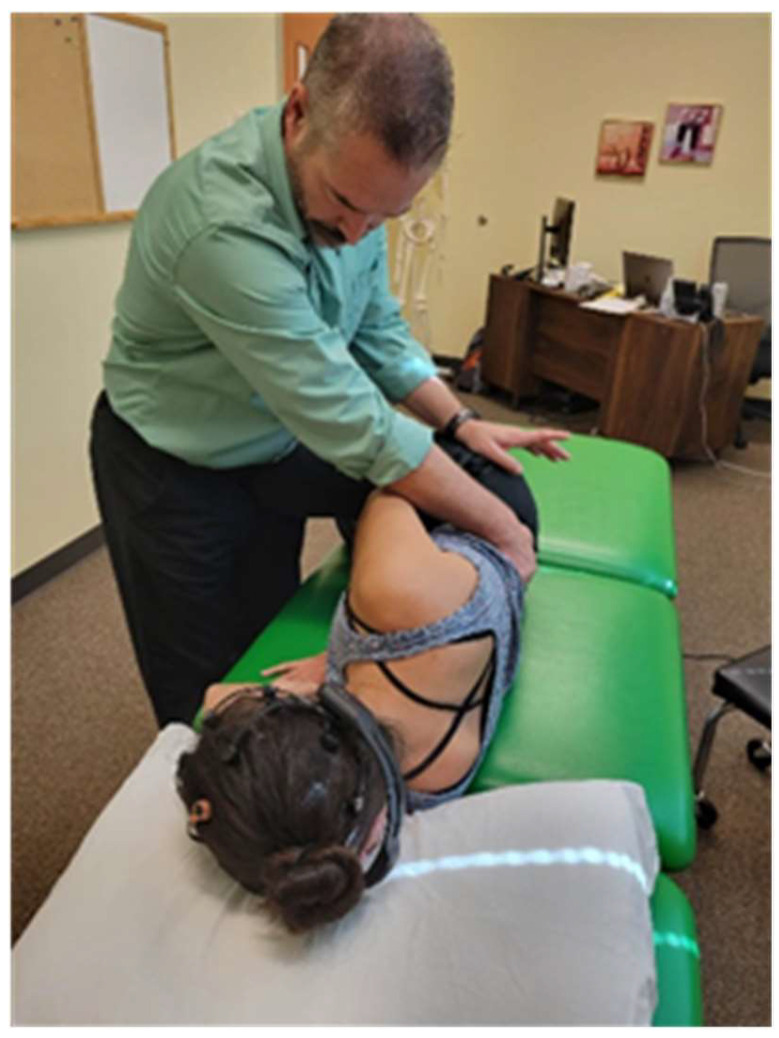
Researcher II uses hip flexion to identify L4–L5 interspinous space.

With the subject in a side-lying position, researcher II used his left middle finger to palpate the interspinous space between the fifth lumbar and first sacral vertebrae. Using passive hip flexion, researcher II could identify the interspinous space between lumbar four and five (Figure 2). 

Next, the subject’s spine was rotated from above to the lumbar four level. This was followed by leg flexion to the same level. In this position, researcher II placed hands on the back of the subject, establishing contact since both the subject’s position on the table and the clinical touch could result in sensory perception by the subject. Furthermore, this sensory input could result in an alteration of brainwave activity. Morita et al. [19] identified that touch by itself can provide a placebo effect and could reduce both anxiety and pain. This concurs with Singh et al. [20], who identified that pleasant touch suppresses low-frequency Alpha/Beta activity in electrodes opposite the side of the touch [20]. Considering this possible placebo effect and the fact that brainwave measurements are performed in real time, the subject might generate physiological artifacts, resulting in brainwave spikes and thus leading to measurement errors.

In this side-lying position, the subject lay still with their eyes closed for three minutes to adapt to any positional and environmental sensory input. The eyes remained closed to avoid brainwave activity in response to ocular or muscular activity [4]. After this three-minute positioning, the first pre-intervention, fifteen-second EEG measure was taken by researcher I. Immediately following this, researcher II carried out a right rotatory HVLA manipulation targeting lumbar four-five, as described by Hartman [21]. Researcher II’s 30 years of manipulative technique experience and fellowships in the American Academy of Orthopaedic Manual Physical Therapists ensured expert consistency in the manipulation. While researcher II maintained contact with the subject, researcher I performed the second EEG measure. After this, researcher II noted the presence or absence of an audible sound during the HVLT manipulation. Next, researcher II moved the subject to independent side-lying and removed the physical touch stimulus. In this position, EEG measures were taken one minute post-intervention and three minutes post-intervention.

## 3. Results

All 44 subjects completed the intervention and measurements. The non-audible group consisted of 21 subjects with a mean age of 24.6, while the audible group consisted of 23 subjects with a mean age of 25.2. Statistical analyses were performed using IBM’s SPSS, version 28.0, statistical software package. A confidence interval of 95% and a significance level of 0.05 were used when analyzing all data. To determine if brainwaves for each frequency band (Theta, Alpha, Beta, and Gamma waves) were normally distributed, the Shapiro–Wilk test of normality was used. This normality test identified no normal distribution of the data with *p* < 0.05; for that reason, the parametric statistics assumptions were not met. 

**Figure 3:**
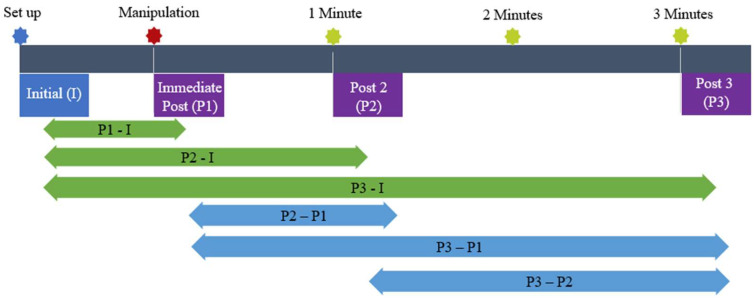
Measurement interval.

The subjects underwent a pre- and post-intervention repeated measures design quasi-experimental study (Figure 3). The Friedman test was used to analyze the data to determine if there were any significant changes in brainwave activity in each frequency band between each electrode’s pre- and post-intervention mean in both the audible and non-audible sound groups. Following this, the Mann–Whitney U test was used for both groups to identify whether significant differences could be found between measures. Next, the percentage of change for the measurement interval for both groups was determined as this might shed light on the difference in the brain’s reactivity for both groups during the measurement phases.

### 3.1. Audible Sound Group

**Figure 4:**
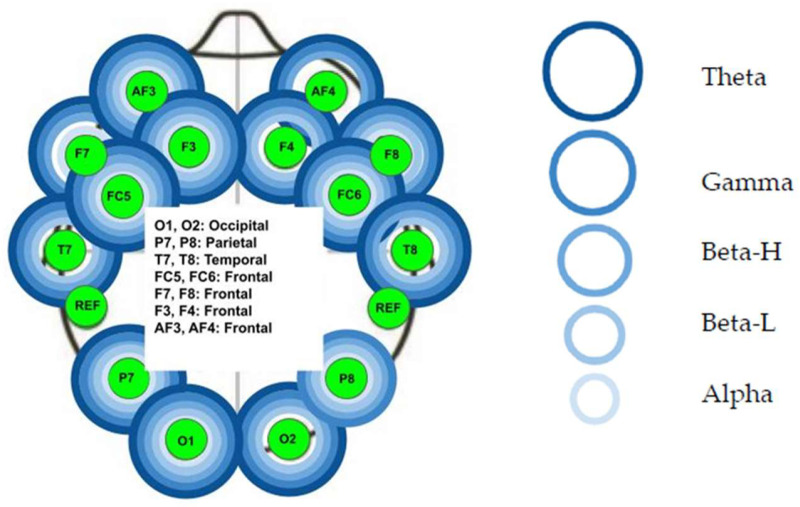
Statistical significance in the audible sound group. The corresponding colored circles for each of the electrodes identify that a significant difference was observed in the corresponding frequency band at that location.

The Mann–Whitney test was used to determine the difference in brainwave activity for each of the five frequency bands between the pre- and post-HVLA manipulation measures. There was a statistically significant difference (*p* < 0.05) at the following electrodes (Figure 4):

1. Left frontal lobe: the AF3 electrode for all five bands (*p* < 0.01), the F7 electrode for all bands (*p* < 0.01) except the Beta L band, the F3 for all five bands (*p* < 0.01), and the FC5 for all five bands (*p* < 0.01).
2. Left temporal lobe: the T7 electrode for all bands (*p* < 0.01) except Alpha band.
3. Left parietal lobe: the P7 electrode for all five bands (*p* < 0.01).
4. Left occipital lobe: the O1 electrode for all five bands (*p* < 0.01).
5. Right occipital lobe: the O2 electrode for all bands (*p* < 0.01) except Alpha band.
6. Right parietal lobe: the P8 electrode for all five bands (*p* < 0.01).
7. Right temporal lobe: not significant (*p* > 0.05).
8. Right frontal lobe: the FC6 electrode for all five bands (*p* < 0.01), the F4 electrode for all bands (*p* < 0.01) except Alpha band, the F8 electrode for all five bands (*p* < 0.01), and the AF4 electrode for the Theta, Gamma, and Beta H bands (*p* < 0.01).

Next, the Wilcoxon test was used to identify where significant changes occurred between each measuring point for each brainwave under each electrode. Table 1 identifies the measurement points and whether there was tactile contact from the researcher with the subjects.

The compared intervals can be found in Figure 3. Four wavelengths in the audible sound group were not significant (*p* > 0.05). These non-significant wavelengths are as follows:

1. Right occipital lobe: O2 Alpha and O2 Beta-L.
2. Right parietal lobe: P8 Alpha.
3. Right temporal lobe: T8 Alpha.

The percentage of significance for each interval was determined for all wavebands under all electrodes for the audible sound group across all wavelengths and is represented in the table below (Table 2). 

### 3.2. Non-Audible Sound Group

**Figure 5:**
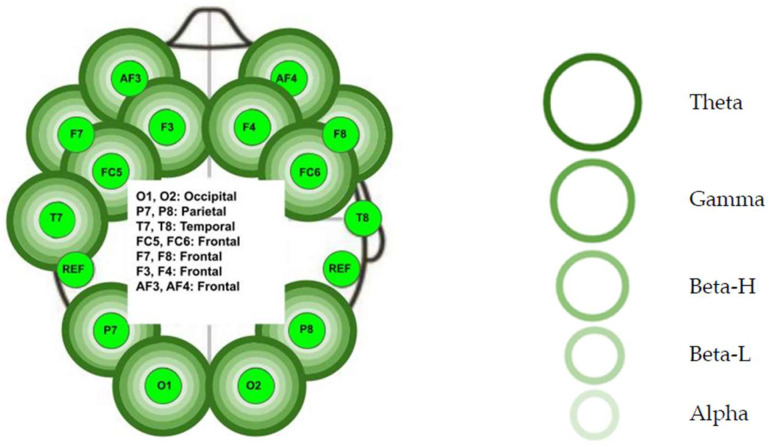
Statistical significance in the non-audible sound group. The corresponding colored circles for each of the electrodes identify that a significant difference was observed in the corresponding frequency band at that location.

The Mann–Whitney test was used to determine the difference in brainwave activity for each of the five frequency bands between the pre- and post-HVLA manipulation measures. There was a statistically significant difference (*p* < 0.05) at the following electrodes (Figure 5):

1. Left frontal lobe: the AF3 electrode for all five bands (*p* < 0.01), the F7 electrode for all five bands (*p* < 0.01), the F3 for all five bands (*p* < 0.01), and the FC5 for all five bands (*p* < 0.01).
2. Left temporal lobe: the T7 electrode for all five bands (*p* < 0.01).
3. Left parietal lobe: the P7 electrode for all five bands (*p* < 0.01).
4. Left occipital lobe: the O1 electrode for all five bands (*p* < 0.01).
5. Right occipital lobe: the O2 electrode for all five bands (*p* < 0.01).
6. Right parietal lobe: the P8 electrode for all bands (*p* < 0.01) except Beta-L (*p* > 0.05).
7. Right temporal lobe: not significant (*p* > 0.05).
8. Right frontal lobe: the FC6 electrode for all five bands (*p* < 0.01), the F4 electrode for all five bands (*p* < 0.01), the F8 electrode for all five bands (*p* < 0.01), and the AF4 electrode for all five bands (*p* < 0.01).

Next, the Wilcoxon test was used to identify where significant changes occurred between each measuring point for each brainwave under each electrode. Figure 3 identifies the compared intervals. Six wavelengths in the non-audible sound group were found to be not significant (*p* > 0.05). These non-significant wavelengths are as follows:

1. Right parietal lobe: P8 Beta-L.
2. Right temporal lobe: T8 Alpha, T8 Beta-H, T8 Beta-L, T8 Gamma, and T8 Theta.

The percentage of significance for each interval was determined for all wavebands under all electrodes for the non-audible group and is represented in the table below (Table 3).

### 3.3. Group Comparison

**Figure 6:**
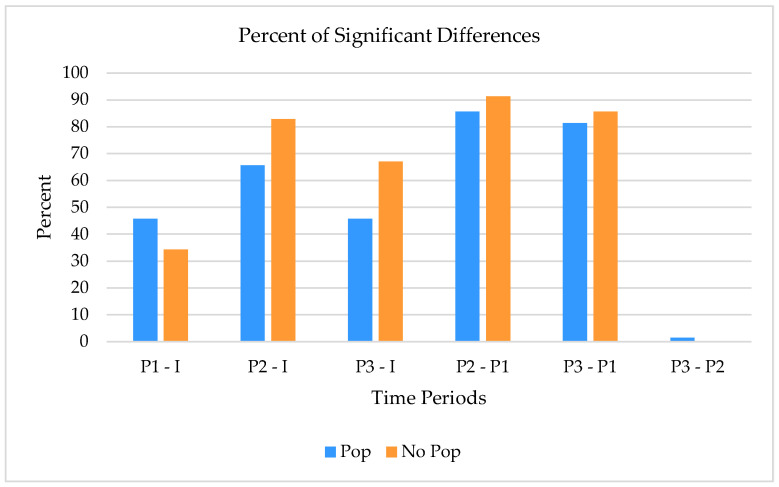
Percentage of significance comparison of the audible and non-audible groups.

When comparing the percentage of significance between both groups for all five measurement intervals, it seems evident that the audible sound group had a higher percentage of change immediately after the HVLT manipulation. Additionally, there is minimal activity in the audible sound group between measures 2 and 3. None was identified for the non-audible sound group (Figure 6). The non-audible pop group had higher significant differences in brainwaves for all measurement periods (*p* < 0.05).

## 4. Discussion

This quasi-experimental repeated measures study aimed to determine if the audible sound often accompanying a lumbar spine HVLA manipulation significantly affects brainwave activity. Forty-four healthy subjects underwent a lumbar 4–5 right rotatory manipulation in a side-lying position. To our knowledge, no other study has evaluated and compared brainwave activity with respect to the presence of an audible or non-audible sound during and following a lumbar HVLA manipulation.

The challenge for this kind of study is that any sensory, audible, and/or tactile input will instantly change brainwave activity. When assessing the difference between each measurement interval between the two groups, both groups showed significant changes at intervals p1–p2 and p1–p3 in greater than 80% of the electrodes and five brainwave types. Since p1 is the HVLA manipulation location, it is possible that an audible sound could have accounted for changes in the audible sound group. One could expect that when audible manipulation sounds are produced, the temporal lobes and primary auditory cortices will be activated [22]. The Emotiv EPOC+ T7 and T8 electrodes measure activity in the temporal lobe. Although it has been hypothesized that audible manipulation sounds should activate this brain region, the results of this study indicate that both groups showed a lack of statistically significant change in the five frequency bands measured under T7 and T8 (*p* > 0.05), indicating a lack of impact of the audible sound on the brain compared to the non-audible HVLA manipulation.

After the p1 measurement point, researcher II removed all contact with the subject by stepping back and removing the manipulation hold position. This action results in a significant reduction in tactile stimulation. A change in tactile stimulation should have immediately impacted brainwave activity. Such a change in brainwave activity has been confirmed previously [19]. Singh et al. [20] demonstrated that Beta waves generally increase, and Alpha waves decrease, when pleasant touch is removed. The findings of our study concur with Singh et al. [20] with respect to the initial observation of brainwave changes. However, any direct impact from removing tactile contact or the HVLA manipulation effect dissipated quickly, and brainwave activity stabilized after two to three minutes. The relative absence of significant changes between p2 and p3 for both groups demonstrated this.

The immediate effect of the HVLA manipulation can be identified by further exploring the difference between the pre- and post-intervention measures for both groups. Alpha waves typically represent a state of relaxation [15]. In the non-audible group, significant changes (*p* < 0.05) in Alpha waves were identified in the right frontal lobe (AF4) and the left temporoparietal lobe (F7). This change would support the immediate relaxation effect after an HVLA manipulation. In the audible sound group, significant changes (*p* < 0.05) were identified in the right and left temporal lobe (T7 and T8), left temporo-occipital lobe (P7), right frontal lobe (AF4), and left temporoparietal lobe (F7 and FC5). The regions affected in the audible pop group are more significant than those in the non-audible group. This implies a more significant relaxation effect from HVLA manipulation with audible sounds.

Gamma waves are typically associated with perception, alertness, and an intake of sensory information [15]. In the non-audible sound group, significant differences (*p* < 0.05) in Gamma waves were identified in the left temporoparietal lobe (F7), left occipital lobe (O1), and right frontal lobe (F4 and AF4). These changes in brainwave activity were primarily in the frontal lobe region and would support an increased alertness and cognitive function after the HVLA manipulation [15]. In the audible sound group, significant changes (*p* < 0.05) were identified in the right frontal lobe (F4), right frontoparietal lobe (F8 and FC6), right parietal lobe (T8), and left temporo-occipital lobe (P7). The audible sound group primarily had changes on the right side and the non-audible group had changes on the left. Although the changes were similar overall, the non-audible group had more frontal lobe changes, possibly indicating greater alertness. Bakker and Miller [23] identified that the frontal lobe plays a role in the placebo effect. If the audible manipulation created a placebo effect, a decrease in frontal lobe Gamma waves would be expected [24,25]. The Gamma brainwave activity changes in both groups’ frontal lobes were similar. Hence, it was concluded that the audible pop was unlikely to produce a “placebo” response in our participants. Despite this finding, one must consider that the audible pop group significantly changed the right temporal lobe brainwave activity. This could reflect the participant’s recollection of the sensation or audible sound caused by the HVLA manipulation.

Theta waves have been related to deep relaxation, memory creation, and learning [15]. In the non-audible group, significant changes in Theta waves were identified in the left parietal lobe (T7), right frontal lobe (F8), left temporo-occipital lobe (P7), right temporo-occipital lobe (P8), left occipital lobe (O1), and right frontal lobe (F4 and AF4). The changes observed in Theta waves were primarily in the frontal lobes. In the audible sound group, significant changes (*p* < 0.05) were identified in the left temporal lobe (T7), left temporoparietal lobe (F7 and FC5), left frontal lobe (F3), right frontal lobe (FC6 and F8), and right temporal lobe. Changes in Theta waves for both groups appear similar, with a high percentage of alterations in the frontal lobes. Our findings concur with Sparks et al. [26], who demonstrated decreased Theta wave activity after an HVLA manipulation in the thoracic spine. Additionally, our observed differences in the frontal lobe could indicate the perception of feeling “better” after an HVLA manipulation. Both groups experienced a similar effect of changes in Theta waves and, thus, relaxation. Relaxation can be associated with a participant’s perception of something good due to previously developed biases and increased relaxation states [20,24].

Beta waves are related to conscious activities, such as creating judgment and decision-making functioning of the brain [15]. In the non-audible group, significant changes (*p* < 0.05) in Beta waves were identified in the left frontal lobe (F3 and F7), left temporo-occipital lobe (P7), left occipital lobe (O1), right temporo-occipital lobe (P8), and right frontal lobe (F4 and AF4). The changes observed in Beta waves have no specific dominance and seem to occur in most of the brain. In the audible sound group, significant changes (*p* < 0.05) were identified in the left frontal lobe (F3), left temporoparietal lobe (F7 and FC5), left temporal lobe (T7), left occipital lobe, right temporal lobe (T8), right temporoparietal lobe (FC6), and right frontal lobe (AF4). Beta waves are associated with activity in the parietal and frontal lobes and are active when conscious thinking and an intensely engaged mind exist. Both groups appear to have similar changes in brainwave activity across the lobes. Singh et al. [20] concluded that Beta wavelengths in the right temporoparietal and frontal regions indicate a subjective perception of pleasant stimuli. They also demonstrated that temporoparietal Beta activity is correlated with a current emotional state. The specific change in Beta activity in the audible sound group could reflect a participant’s perception of audible sound during the HVLA manipulation. 

Based on our findings, the clinical considerations are that any audible sounds that might coincide with an HVLT manipulation are likely not contributing to any manipulation placebo effect. Therefore, the clinical significance of this study is that audible sounds should not be used to determine the effectiveness of HVLT lumbar manipulations. Thrust manipulation significantly affects brainwave activity regardless of audible sounds and enhances an overall sense of relaxation. Although the short-term effect of this relaxation is somewhat more widespread for the audible sound group, the true clinical application of this phenomenon is not known currently. Future research should investigate this effect over time and relate the effect to other central nervous system factors, such as the autonomic nervous system.

### Limitations

This study was challenged by the occasional difficulty maintaining strong contact between the Emotiv EPOC+ electrodes and the skull to prevent signal generation caused by artifacts. The difficulty was particularly prevalent when researcher II maneuvered the participant into the correct position on the treatment table to perform the HVLA manipulation. To maintain optimal contact with the electrodes, researcher II rotated the thoracic spine less, reducing the head movement on the pillow. In contrast, the lumbar spine and lower extremities moved more. Another limitation was that only 44 participants completed the study protocol, limiting the study results’ generalizability. Finally, although the data collection location was chosen to limit audible noises, there was an occasional noise factor that could not be controlled.

## 5. Conclusions

When managing lower back pain, HVLA manipulation is often used to manage pain and mobility. During the HVLA manipulation, audible sounds are frequently generated. Although the exact physiological mechanism regarding the sounds remains elusive, practitioners and patients tend to regard this as a sign of a positive outcome. The results of this study identified that subjects in the audible sound group appeared to have more initial changes in Alpha waves in the frontal lobe. Meanwhile, the brainwave changes in Beta, Gamma, and Theta frequencies between the audible and non-audible groups were similar. The lack of brainwave response differences between groups further demonstrated that there was likely no direct placebo effect from the audible sound. Additionally, based on this study, audible sounds cannot be used to determine the effectiveness of the centralized effect of HVLA lumbar manipulation. This study could not identify a benefit from the audible sound during an HVLA manipulation of the participants. Further studies are warranted to correlate possible audible manipulation sounds with clinical effectiveness and brainwave activity. Additionally, future studies should evaluate the effect of thrust manipulation in a symptomatic subject population and investigate how pain might influence brainwave activity.

## Figures and Tables

**Table 1:** Measurement points with position and contact.

	Name	Time	Position	Tactile Touch
I	Initial	Immediately before manipulation	Manipulation position	Present
P1	Post 1	Immediately after manipulation executed	Manipulation position	Present
P2	Post 2	1 min after manipulation	Relaxed side-lying	Absent
P3	Post 3	3 min after manipulation	Relaxed side-lying	Absent

**Table 2:** Percentage of significance in summation for all five wavebands under all electrodes per measurement interval: pop group.

Audible Sound Group	
Intervals	% of Significance
P1-I	45.7%
P2-I	65.7%
P3-I	45.7%
P2–P1	85.7%
P3–P1	81.4%
P3–P2	1.4%

**Table 3:** Percentage of significance in summation for all five wavebands under all electrodes per measurement interval: non-pop group.

Non-Audible Sound Group	
Intervals	% of Significance
P1–I	34.3%
P2–I	82.9%
P3–I	67.1%
P2–P1	91.4%
P3–P1	85.7%
P3–P2	0%

## Data Availability

The original contributions presented in the study are included in the article; further inquiries can be directed to the corresponding author.
